# *Mycobacterium avium* Complex Infection as a Rare Cause of Cerebral Mass Lesion and IRIS in a Patient With AIDS: Case Report and Review of the Literature

**DOI:** 10.1093/ofid/ofab450

**Published:** 2021-10-30

**Authors:** Courtney Lane-Donovan, Emma Bainbridge, John Szumowski, Andrew D Kerkhoff, Michael J Peluso

**Affiliations:** 1 Department of Neurology, University of California San Francisco, San Francisco, California, USA; 2 Division of HIV, Infectious Diseases and Global Medicine, Department of Medicine, Zuckerberg San Francisco General Hospital and Trauma Center, University of California San Francisco, San Francisco, California, USA

**Keywords:** HIV, IRIS, MAC, space-occupying lesion, universal broad-range PCR

## Abstract

A patient with advanced HIV/AIDS presented with a brain abscess. While brain biopsy culture and pathology were unrevealing, universal broad-range polymerase chain reaction (uPCR) demonstrated *Mycobacterium avium* complex (MAC). We review the clinicopathologic characteristics of MAC brain abscesses and highlight the effectiveness of uPCR as a diagnostic tool in partially treated infections.

A 49-year-old man with advanced HIV/AIDS presented to the emergency department after he was found down in the setting of methamphetamine and fentanyl intoxication. A noncontrast computed tomography (CT) scan of the head showed a 0.7-cm left parietal mass (Figure 1A). Brain magnetic resonance imaging (MRI) with and without contrast showed a 0.5-cm rim-enhancing lesion in the left parietal lobe with central restricted diffusion and surrounding vasogenic edema (Figure 1A) concerning for parietal abscess.

**Figure 1.**
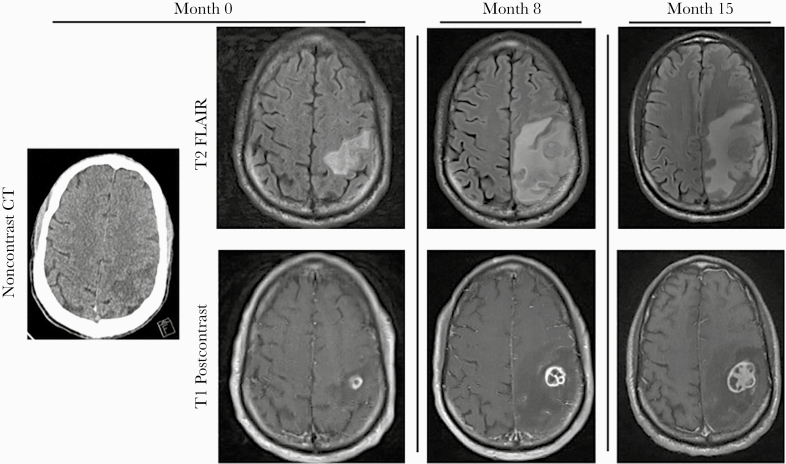
A, Noncontrast head CT, T2 FLAIR precontrast MRI, and T1-weighted postcontrast MRI from initial presentation. B, T2 FLAIR precontrast MRI and T1-weighted postcontrast MRI after discontinuing ART and MAC therapy. C, T2 FLAIR precontrast MRI and T1-weighted postcontrast MRI following completion of prednisone taper. Abbreviations: ART, antiretroviral therapy; CT, computed tomography; FLAIR, fluid-attenuated inversion recovery; MAC, *Mycobacterium avium* complex; MRI, magnetic resonance imaging.

The patient was diagnosed with HIV infection in 1989 and had been intermittently adherent to antiretroviral therapy (ART). He had been diagnosed with *Streptococcus pneumoniae* tricuspid valve endocarditis 4 months prior and completed treatment with 6 weeks of intravenous vancomycin. Two months prior, he was diagnosed with cytomegalovirus (CMV) retinitis after being off ART for >1 year (CD4+ T-cell count <10 cells/µL, plasma HIV RNA 52 893 copies/mL). A CT scan of the head at this time had been unremarkable. He initiated ART with dolutegravir, abacavir, and lamivudine (DTG/ABC/3TC). However, he was inconsistently adherent, and he was not taking valganciclovir or prophylaxis against *Pneumocystis jirovecii* or *Mycobacterium avium* complex (MAC), which were indicated for his low CD4+ T-cell count and inconsistent ART use. His social history was notable for injection drug use and housing instability.

His CD4+ T-cell count at presentation was 63 cells/µL, with plasma HIV RNA 11 006 copies/mL. He was started on empiric vancomycin and ceftriaxone for a brain abscess. His therapy did not include toxoplasmosis coverage given a prior negative toxoplasma immunoglobulin G (IgG). Negative studies included bacterial blood cultures, repeat serum *Toxoplasma* IgG, cryptococcal antigen, *Coccidioides* immunodiffusion, and rapid plasma reagin (RPR). A lumbar puncture revealed 2 white blood cells, 0 red blood cells, and normal protein and glucose in the cerebrospinal fluid (CSF). CSF bacterial, fungal, and AFB cultures, CMV PCR, cryptococcal antigen, and *Coccidioides* complement fixation testing were negative. Transthoracic echocardiogram demonstrated a stable tricuspid vegetation without new findings. A brain biopsy was not pursued at this time as the patient was neurologically intact and the lesion was near the motor strip. After 3 weeks, he was discharged to a skilled nursing facility (SNF) to complete an 8-week antibiotic course for presumed pyogenic abscess, and he was restarted on DTG/ABC/3TC and valganciclovir.

A few days after discharge, he presented with blurry vision and right-sided neck swelling. Ophthalmologic exam found no abnormalities. MRI revealed that his brain lesion had increased to 1.5 cm in diameter. Additional CSF studies, including flow cytometry for leukemia and lymphoma markers, were negative. Ultrasound of the right neck showed a 4.3-cm fluid collection with reactive cervical lymph nodes. The collection was drained, with resultant growth of MAC on culture. Sputum cultures also grew MAC. A brain biopsy was again deferred due to surgical risk, and the brain lesion was also presumed to be due to MAC. He was discharged to an SNF on ethambutol, rifabutin, and azithromycin for treatment of disseminated MAC. After observing no improvement in his brain abscess size over several months, moxifloxacin was added to his anti-MAC regimen given its good central nervous system (CNS) penetration. He was lost to follow-up shortly thereafter. He had a brief presentation to the emergency department after a drug overdose, for which a CT chest was obtained. The imaging showed multifocal nodular consolidations felt to be consistent with aspiration given the clinical setting, and he was discharged.

Eight months after his initial admission, he was readmitted with profound sensory ataxia of his right arm following self-discontinuation of ART and anti-MAC therapy. His CD4+ T-cell count was 183 cells/µL and plasma HIV RNA was 15 000 copies/mL. MRI of the brain showed interval increase in the size of the multiloculated, rim-enhancing parietal lobe lesion to 1.8 cm (Figure 1B). Given his clinical and radiographic worsening, a brain biopsy was pursued. Pathology showed reactive changes and lymphohistiocytic inflammation, but no organisms were identified by staining. Bacterial, fungal, and mycobacterial cultures of his brain biopsy were negative, as were bacterial, fungal, and mycobacterial universal broad-range PCRs (uPCRs). Given that he had been intermittently adherent with his anti-MAC regimen, his working diagnosis remained the same, and he was restarted on DTG/ABC/3TC and his 4-drug anti-MAC regimen (ethambutol, rifabutin, azithromycin, moxifloxacin).

One month later, he developed focal seizures and right arm weakness. A repeat noncontrast head CT showed increased abscess size, surrounding edema, and midline shift. He underwent abscess incision and drainage, which yielded a scant quantity of purulent fluid. One week later, the mycobacterial uPCR on this sample was sent to the University of Washington Molecular Diagnostics microbiology lab and returned positive for MAC, with analytical sensitivities (ie, the number of genomic copies required for a positive result) for 16S rRNA of 100, heat shock protein (hsp65) of 5, and RNA polymerase β subunit (rpoB) of 5 [1]. Bacterial, fungal, and AFB cultures were again negative. He was discharged with a prolonged anti-MAC treatment course.

He returned 3 weeks after discharge with altered mental status. The parietal abscess was stable in size on contrast-enhanced CT, but there was marked surrounding edema, raising concern for paradoxical MAC IRIS. In addition to his ART and anti-MAC therapy, the patient was started on prednisone. His mental status improved, and following completion of a 3-month prednisone taper, his right upper extremity strength improved as well. His CD4+ T-cell count rose to 253 cells/µL and plasma HIV RNA was 63 copies/mL. Despite symptomatic improvement, MRI of the brain demonstrated enlargement of the abscess, worsening edema, and midline shift (Figure 1C, overall course shown in Figure 2). These inflammatory changes were attributed to IRIS, prompting re-initiation of prednisone.

**Figure 2.**
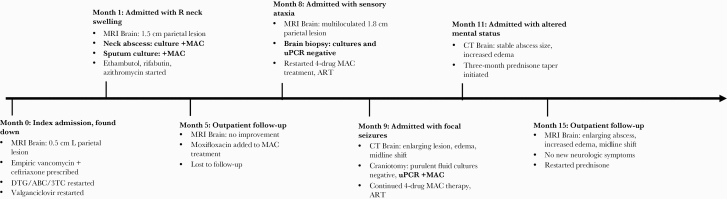
Timeline of events. Abbreviations: ART, antiretroviral therapy; CT, computed tomography; DTG/ABC/3TC, dolutegravir, abacavir, and lamivudine; MAC, *Mycobacterium avium* complex; MRI, magnetic resonance imaging; uPCR, universal broad-range polymerase chain reaction.

Nearly 1 year after diagnostic confirmation, he remains intermittently adherent to 4-drug anti-MAC treatment and prednisone. He has no new neurologic symptoms, but the size of his brain abscess on interval head CT remains unchanged. Given his clinical stability and the risk of repeated surgical procedure, a repeat incision and drainage has not been performed, and no further diagnostic workup has been conducted.

## DISCUSSION

We report a case of MAC brain abscess in a patient with advanced HIV/AIDS that was diagnosed by universal broad-range PCR. MAC is the most common nontuberculous mycobacterium identified in the United States, and the best-described species are *M. avium* and *M. intracellulare.* These organisms are transmitted through inhalation and ingestion and cause infections in both immunocompromised and immunocompetent hosts, with distinct clinical manifestations in each group [2]. Advanced HIV/AIDS represents the most common risk factor for MAC infection [3], and in patients with HIV, the most common presentations are disseminated MAC and localized lymphadenitis. Rarely, MAC may cause other forms of infection, including skin, soft tissue, bone, liver, and spleen disease [2, 4]. In contrast, in immunocompetent patients, MAC typically causes chronic, isolated pulmonary infections.

Our patient was diagnosed with suppurative cervical lymphadenitis due to MAC as well as a solitary MAC brain abscess, representing both a typical presentation of MAC infection in advanced HIV/AIDS and a rarely described manifestation involving the CNS. In our review of the literature, CNS MAC infections are seen primarily in immunocompromised patients (Table 1). Twenty cases of MAC brain abscess have been reported (including the current case), 17 of which occurred in men, with an average age (range) at diagnosis of 45 (33–69) years [4–22]. More than half of patients were diagnosed with a MAC brain abscess in the absence of known pulmonary or disseminated MAC infection (Table 1). Eleven cases occurred in patients with HIV [5–12, 22]. In these cases, CD4+ T-cell count ranged from 2 to 210 cells/µL (median, 31 cells/µL), and only 2 patients had an undetectable viral load at the time that the brain abscess was identified [4]. In 4 cases, the patient had previously completed a course of therapy for disseminated MAC, was continued on ART, and subsequently presented with MAC brain abscesses that were attributed to MAC IRIS up to 2 years after ART initiation [9, 11, 12, 14].

**Table 1.** Demographics, HIV Status, Clinical and Radiographic Features, Treatment, and Outcomes of Patients With MAC Brain Abscesses

	Author (Year)	Age	Sex	Immunocompromising Condition	Presenting Symptoms	No. of Lesions (Largest Size)	Location of Brain Lesions	How Was Diagnosis Made?	Histopathology Findings From Brain Biopsy	Additional MAC Investigations, Including Pertinent Negatives	Treatment (Duration)	Outcome
1.	Murray (2001)	35	M	HIV-positive (CD4 210 cells/µL), known HIV diagnosis, on ART >2 y	Headaches, dizziness, fever, emesis	Single (3 cm)	Frontal	Brain Bx→AFB smear and Cx pos.	Granulomatous inflammation, giant cells, focal necrosis	History of disseminated MAC treated 1 y; therapy stopped >1 y before presentation	RFB, INH, PZA, EMB, CLR [6 wk]→ RFB, INH [NR]	Alive after 24 mo
2.	Berger (2004)	40	M	HIV-positive (CD4 31 cells/µL), known HIV diagnosis, recent ART restart	Seizures	Single (2 cm)	Occipital	Brain Bx→ AFB smear and Cx pos.	Spindle cells in fascicle pattern	History of disseminated MAC treated 2 y; therapy stopped >1 y before presentation	CLR, EMB, CIP (6 mo)→AZM, EMB (NR)	Alive at 10 mo, with resolution of lesion on CT
3.	Kishida (2008)	51	M	HIV-positive (CD4 26 cells/µL), new HIV diagnosis, off ART, diabetes, HCV	Confusion, loss of consciousness	Multiple (NR)	Frontal, parietal, temporal	Postmortem brain Bx→ histological (granulomas; Cx+PCR neg.)	Granulomatous reaction with lymphocytes, histiocytes, fibrous tissue, necrosis	MAC isolated from gastric lavage; AFB sputum and blood Cx neg.	KAN, CLR, RFB, EMB→ EMB, INH, LFX, AMK (~10 mo at time of death)	Clinical deterioration and death 4 mo after starting ART (but also had CNS lymphoma)
4.	Verma (2009)	33	M	HIV-positive (CD4 2 cells/µL), known HIV diagnosis, off ART	Progressive weakness, lethargy, dysphagia, speech slowing, left-sided weakness	Multiple ring-enhancing (NR)	Frontal, parietal, occipital	Brain Bx→ AFB smear and Cx pos.	NR	MAC isolated from blood, sputum, lymph node bx	EMB, RIF, AMK (NR)	Deceased; transitioned to palliative care
5.	Fortin (2012)	36	M	HIV-positive (CD4 170 cells/µL), known HIV diagnosis, on ART 2 y	Headache, expressive aphasia	Multiple ring-enhancing (1.3 cm)	Temporal, tempero-parietal	Brain Bx→ AFB smear and Cx pos.	Reactive gliosis, lymphocyte infiltration, foamy monocytes; no giant cells or granulomas	History of disseminated MAC treated 1 y; therapy stopped >1 y before presentation	AZM, EMB, RFB (>21 mo)	Resolution of lesions after 10 mo; temporal lesion returned 1 mo after stopping treatment; alive and head CT normal at 22 mo
6.	Karne (2012)	42	F	HIV-positive (CD4 14 cells/µL), known HIV diagnosis, off ART	Confusion, left hemiplegia	Single ring-enhancing (5 cm)	Fronto-parietal	Brain Bx→ AFB smear and Cx pos.	NR	NR	CLR, EMB (NR)	Improved CT brain imaging after 1 wk; no follow-up reported
7.	Lee (2013)	23	M	HIV-positive (CD4 70 cells/µL), known HIV diagnosis, on ART >1 y	Progressive paraplegia, low back pain	Multiple (NR)	Bilateral cerebrum, cerebellum, spinal cord	Lumbar puncture→ Cx pos.	NR	History of disseminated MAC treated 1 y; therapy stopped 2 mo before presentation; AFB blood Cx pos during admission	LFX, CLR, EMB, RFB, steroids (NR)	Alive and stable on therapy 1 mo later
8.	Begley (2015)	47	M	HIV-positive (CD4 22 cells/µL), new HIV diagnosis, off ART	Weakness, headache, confusion, blurry vision, weight loss	Multiple ring-enhancing (NR)	Cerebellar, frontal, parietal, temporal, thalamus	Brain Bx→ AFB smear and Cx pos.	Necrotizing tissue; no granulomas	AFB blood and bone marrow Cx neg.	CLR, CIP [→RIF], EMB (NR)	Alive after 6 mo, resolution of lesion on CT at 6 mo
9.	Muzaffar (2017)	59	M	HIV-positive (CD4 20 cells/µL), known HIV diagnosis, poor ART adherence, diabetes, ESRD, HCV	Confusion	Single (9 mm)	Temporal	Brain Bx→ histo-pathological characteristics (AFB present)	Spindle cells	NR	RFB, EMB, ERY (6 mo)	NR
10.	Goodman (2020)	45	M	HIV-positive (CD4 108 cells/µL), known HIV diagnosis, poor ART adherence	Confusion, hallucinations	Multiple (8 mm)	Parietal, temporal	Brain Bx→ universal PCR pos. (Cx not performed)	Nodular granulomata, lympho-plasmacytoid cells, histiocytes, rare giant cells, micronecrosis	MAC isolated from sputum	RIF, INH, PZA, EMB, CLR, [→AZM], steroid taper (NR)	Alive at 6 mo, no repeat imaging reported
11.	Present case (2021)	49	M	HIV-positive (CD4 63 cells/µL), known HIV diagnosis, recent ART restart, HCV	Loss of consciousness	Single ring-enhancing (3.5 cm)	Parietal	Brain Bx→ universal PCR pos. (AFB smear and Cx neg.)	Lymphohistiocytic infiltration with extensive reactive astrogliosis	MAC isolated from sputum and lymph node	AZM, RFB, EMB, MFX, steroids (>22 mo)	Alive at 23 mo, MRI unchanged after 15 mo (ongoing adherence challenges)
12.	Uldry (1992)	31	F	HIV-negative (“normal CD4 count”), previously healthy	Headaches, ataxia, confusion, emesis	Single (NR)	Temporal	Brain Bx→ AFB smear and Cx pos.	Giant multinuclear cells, granulomas	AFB blood and CSF Cx neg.	CIP, AMK, EMB, CFZ, RIF, prednisone (~10 mo after time of death)	Died after 10 mo as complication of MAC
13.	Dickerman (1996)	38	M	HIV-negative, sarcoidosis (prior long-term steroid use), INF-gamma and TNF-alpha deficiencies	Seizure, ataxia	Multiple (NR)	Frontal, parietal, cerebellum	Brain Bx→ AFB Cx pos.	Encapsulated abscess, with surrounding reactive astrocytosis	AFB blood, BAL, bone marrow, and sputum Cx neg.	RIF, EMB, CLR (NR), →progressed requiring surgical resection	Alive 6 mo after surgery, with resolution of lesions at 6 mo on MRI
14.	Morrison (1999)	38	M	HIV-negative (CD4 90 cells/µL), sarcoidosis (on steroids), chronic neutropenia	Headaches	Multiple (NR)	Frontal, parietal, cerebellum	Brain Bx→ AFB smear and Cx pos.	Spindle cells, lymphocytes, plasma cells, and PMNs	NR	AMK, CLR [→AZM], EMB, RIF (NR)	Acutely improved
15.	Di Patre (2000)	50	F	HIV-negative, SLE (on prednisone and azathioprine)	Enlarging scalp mass	Single, meningioma (NR)	Frontal-region	Brain Bx→ AFB smear, culture pos.	Dural-based spindle cells, fascicular AFB-laden histiocytes; no caseating necrosis or giant cells	NR	None (surgical removal)	NR
16.	Sadek (2008)	63	M	HIV-negative (CD4 220 cells/µL), sarcoidosis (not on immunosuppressives)	Headaches, word-finding difficulties	Single, ring-enhancing (NR)	Frontal	Brain Bx→ AFB smear positive (unclear culture result)	Spindle cell pseudotumor formation	NR	EMB, CLR (11 mo)	Alive after 12 mo, with imaging resolution of lesion at 12 mo
17.	Arkun (2012)	52	M	HIV-negative (CD4 175 cells/µL), sarcoidosis (not on immunosuppressives)	Headaches, dizziness, gait disturbance and falls	Single ring-enhancing (NR)	Cerebellum, tempero-occipital	Brain Bx→ AFB smear, MAC DNA probe pos.	Spindle cells, lymphocytes, plasma cells, single giant cell	NR	NR	NR
18.	Chowdhary (2015)	40	M	HIV-negative (CD4 515 cells/µL), previously healthy	Headaches, blurry vision, diplopia, emesis	Single ring-enhancing (3.4 cm)	Frontal	Brain Bx→ AFB smear and Cx pos.	Granulomatous inflammation, necrosis	MAC isolated from sputum; AFB blood Cx neg.	CLR, EMB, RIF (12 mo)	Alive after 12 mo, with resolution of lesion at 8 mo on MRI
19.	Ismail (2015)	69	M	HIV-negative, sarcoidosis (on prednisone >2 y)	Headaches, seizures	Single (NR)	Temporal	Brain Bx→ AFB smear and MAC PCR pos.	Dural-based, cellular nodules, spindle cells, lymphocytes, and occasional PMNs	MAC previously isolated from sputum on several occasions	EMB, RIF, MOX (2 y)	Alive after 2 y, resolution of lesion on MRI after 2 y
20.	Kotecha (2018)	54	M	HIV-negative, diabetes	Confusion	Multiple (NR)	Bilateral cerebrum	Brain Bx→ AFB smear positive (unclear Cx result)	NR	MAC isolated from sputum 1 y previously (not treated); lung bx AFB smear positive during admission	CMB, RIF, CLR (NR)	Acutely improved

Abbreviations: AFB, acid fast bacilli; AMK, amikacin; ART, antiretroviral therapy; AZM, azithromycin; BAL, bronchoalveolar lavage; Bx, biopsy; CFZ, clofazimine; CLR, clarithromycin; CSF, cerebrospinal fluid; CT, computed tomography; Cx, culture; EMB, ethambutol; ERY, erythromycin; INH, isoniazid; KAN, kanamycin; LFX, levofloxacin; MAC, *Mycobacterium avium* complex; MOX, moxifloxacin; MRI, magnetic resonance imaging; neg, negative; NR, not reported; PMN, polymorphonuclear leukocytes; pos, positive; PZA, pyrazinamide; RFB, rifabutin; RIF, rifampicin; SLE, systemic lupus erythematosus.

The clinical presentations of patients with MAC brain abscesses are similar to other space-occupying lesions. The majority of patients experienced headache and other nonspecific neurologic complaints, including dizziness, nausea/vomiting, altered mental status, and gait instability. Five patients experienced seizures, and specific neurologic deficits were described depending on the location of the abscesses, including focal weakness, expressive aphasia, ataxia, and diplopia [5, 16]. In contrast to pyogenic abscess, fever was described in only 1 case report [12].

Radiographically, there is no clear predilection for a specific location within the brain, as MAC abscesses have been identified in all lobes, consistent with hematogenous spread. Multiple lesions were demonstrated on neuroimaging in 9 of the previously reported cases, whereas a solitary lesion was identified in 11 cases (Table 1). Ring enhancement, mass effect, and edema are frequently described, but leptomeningeal enhancement is less common. In 1 particularly aggressive case, a 40-year-old immunocompetent man had 2 frontal lobe abscesses with erosion through the orbital roof [4]. Based on the pathologic and radiographic features of MAC brain abscesses, the differential diagnosis for this entity is wide. In immunocompromised hosts with space-occupying brain lesions, MAC brain abscesses should be considered along with pyogenic bacterial abscesses and intracranial infections due to *Mycobacterium tuberculosis*, *Nocardia*, *Rhodococcus*, *Cryptococcus*, *Toxoplasma*, *Histoplasma*, *Aspergillus*, and *Treponema pallidum*, as well as primary CNS lymphoma, other neoplasms, and autoimmune conditions.

MAC brain abscesses are challenging to diagnose, requiring tissue sampling. Though peripheral MAC infections classically are associated with granulomatous inflammation, similar histologic changes are rarely identified in MAC brain abscesses. The majority of reported cases have been characterized by “spindle cell pseudotumor,” in which spindle-shaped histiocyte proliferation results in tumor-like lesions that contain mycobacteria. In a literature review of 51 cases of spindle cell pseudotumor, 26 (51%) had HIV and 24 (47%) had MAC infections [23]. In the reported cases of MAC brain abscess in which tissue could be obtained, AFB cultures from the brain biopsy frequently grew MAC, while CSF fluid cultures were negative or not reported, including ventricular fluid from a patient who developed hydrocephalus due to abscess blockage of the fourth ventricle [14, 16].

Our case was unusual in its pyogenic appearance, as opposed to the mass-like pseudotumor phenotype. As the patient had received extensive empiric therapy previously, the diagnostic yield of biopsy tissue by traditional methods was limited. Thus, a sample of the purulent material was sent for uPCR, which confirmed the diagnosis. In bacterial and non-TB mycobacterial uPCR, broad-range primers for 16S rRNA are used that amplify all bacterial 16S rDNA fragments, which can subsequently be sequenced to provide species identification [24]. Additionally, hsp65 and rpoB are similarly used to amplify and sequence mycobacterial DNA, which again confirmed MAC in our case. Though a prior case used mycobacterial uPCR to speciate acid-fast bacilli seen on brain biopsy, ours is the first reported case of MAC brain abscess identified solely through uPCR [22]. In this case, we suspected that his cultures were negative given the fastidious nature of MAC and the fact that it had already been partially treated for >1 month. It is important to note that pathogen DNA could persist after mycobacterial eradication, lending uncertainty about the degree to which his brain mass was due to replicating mycobacteria as opposed to an inflammatory response to nonviable organisms. Given his profound immunocompromise and plan to use corticosteroids to address suspected IRIS, we elected to conservatively treat with antimicrobials targeting MAC despite negative AFB cultures.

As a diagnostic tool, uPCR is most likely to be positive in samples with evidence of inflammation or purulence. A recent retrospective analysis showed that uPCR is clinically significant in 10% of cases and management-changing in 4% [25]. Of particular relevance, if a positive result is found on mycobacterial uPCR, it is clinically significant for 92% of cases, compared with 50% for bacterial and fungal uPCR results [25]. Additionally, while AFB cultures can take weeks to become positive, the turnaround time for universal PCR is shorter, which may expedite diagnosis. Of note, our case required 2 samples to be sent for uPCR, suggesting that multiple samples may increase the diagnostic yield of uPCR, though this remains to be studied.

Treatment of MAC brain abscess is not well defined. The majority of cases have been treated with a 3-drug regimen of a macrolide (azithromycin or clarithromycin), ethambutol, and rifabutin, extrapolating from treatment guidelines for disseminated MAC [2]. Macrolides and ethambutol are thought to reach sufficient CSF concentrations only in the setting of meningeal inflammation, and it is worth noting that many of these MAC brain abscesses have been recognized in the absence of meningitis [26]. Furthermore, rifamycin concentrations may not achieve the minimum inhibitory concentration for some mycobacterial strains in the CNS [27]. These concerns prompted us to intensify our patient’s MAC therapy with moxifloxacin when his brain abscess did not demonstrate radiological improvement [28]. Similar approaches have been considered for CNS tuberculosis [29]. Fluoroquinolones (ciprofloxacin, moxifloxacin) [5, 10, 11, 14, 16, 19] and aminoglycosides (amikacin and kanamycin) [7, 10, 15, 16] have also been employed in other reported cases of MAC brain abscesses. In the cases that provide details of the treatment course, antibiotics were provided for 6 to 24 months, and the majority of patients experienced clinical and radiographic improvement with these regimens (Table 1). Patients with HIV were all continued on ART.

The phenomenon of paradoxical IRIS, or the worsening of clinical or subclinical infections after the initiation of ART and partial restoration of immune function, has been seen with MAC infection, where it typically presents as lymphadenitis with or without suppuration [30]. The optimal duration of steroids for CNS MAC IRIS is unknown. Brief (4–8-week) steroid courses are recommended for treatment of MAC IRIS in patients without CNS features. In 3 prior cases of CNS MAC abscess in which worsening inflammation or deterioration following ART initiation occurred suggesting possible paradoxical MAC IRIS, steroids were initiated [14, 22]. For TB IRIS involving the CNS, gradual steroid tapers guided by clinical symptoms over 2 to 3 months are recommended [2]; this approach informed our prolonged taper.

Though MAC brain abscesses are rare, this diagnosis should be considered, especially in immunocompromised patients with abscesses that are not responsive to empiric antibiotic therapy and who have known concomitant MAC disease in other parts of the body. The diagnosis can be challenging to make given variable radiographic and histologic features, and uPCR represents an available complementary tool to increase diagnostic yield.
